# Bleeding Brunner’s Gland Hyperplasia of the Duodenum: A Case Report With a Review of the Japanese Literature

**DOI:** 10.4021/gr532e

**Published:** 2013-05-03

**Authors:** Tateki Yamane, Takayuki Ishii, Akira Umeda, Shigeharu Takagi, Hitoshi Shimao

**Affiliations:** a Division of Gastroenterology, Department Of Internal Medicine, The International University of Health and Welfare, Shioya Hospital, Japan; b Sanikukai Family Clinic, Japan; c Division of Pulmonology, Department Of Internal Medicine, The International University of Health and Welfare, Shioya Hospital, Japan; d Division of Neurology, Department Of Internal Medicine, The International University of Health and Welfare, Shioya Hospital, Japan; e Department of Surgery, The International University of Health and Welfare, Shioya Hospital, Japan

**Keywords:** Duodenal Brunner’s gland hyperplasia, Gastrointestinal bleeding, Endoscopic resection

## Abstract

A man taking antithrombotic agents was admitted because of melena. Upper gastrointestinal endoscopy revealed a large, pedunculated polyp bleeding from erosions on its top. The polyp was endoscopically resected, and histopathologically diagnosed as Brunner’s gland hyperplasia. It is commonly encountered as a small, raised lesion, but may enlarge or bleed. We report this case, with a review of the Japanese literature and discussion of the mechanism of bleeding.

## Introduction

Duodenal Brunner’s gland hyperplasia is a common, elevated lesion, which is small and causes no problems. However, rarely, it enlarges and bleeds. We report a patient receiving antithrombotic therapy who was found to have large and hemorrhagic Brunner’s gland hyperplasia of the duodenum during evaluation for melena, and underwent endoscopic resection of the lesion. In addition, we review the Japanese literature for hemorrhagic cases, and discuss the mechanism of bleeding.

## Case Report

A 61-year-old man was admitted with a 1-week history of melena. He had undergone an operation of valve replacement and coronary artery bypass grafting for aortic valve stenosis and angina at the age of 59 years, and had been taking warfarin and low dose aspirin (LDA) after surgery.

Physical examination showed tachycardia and anemia. Blood tests revealed a low hemoglobin level of 7.4 g/dL and prolonged PT-INR of 1.84 (because of warfarin therapy).

**Figure 1:**
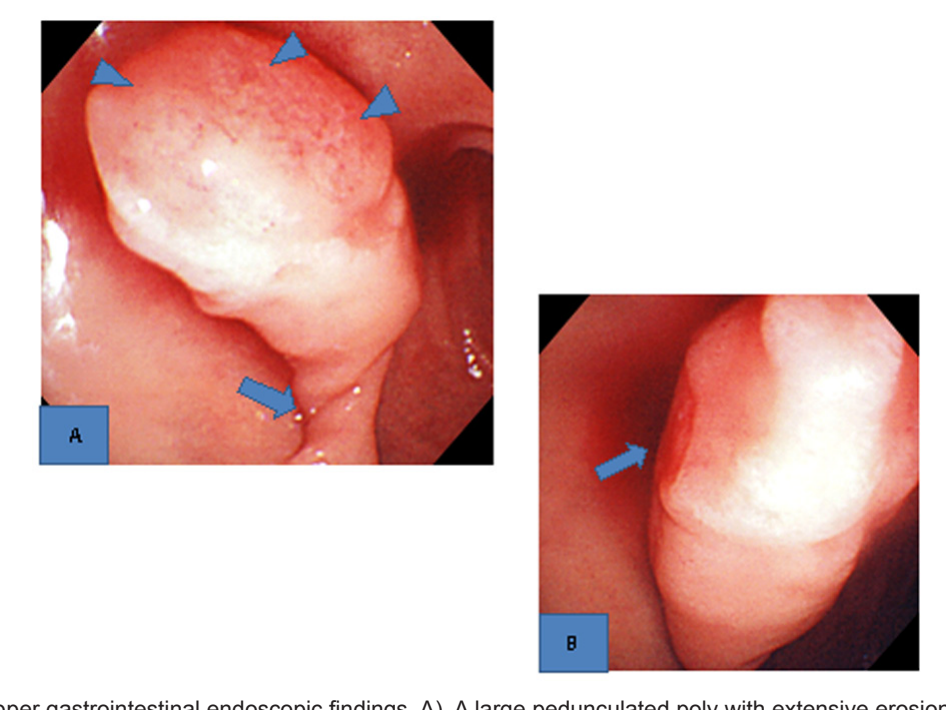
Upper gastrointestinal endoscopic findings. A). A large pedunculated poly with extensive erosions on the top (arrowheads) and a twisted pedicle (arrow) was found in the duodenal bulb. B). Oozing hemorrhage from the erosions on the top was seen (arrow).

Upper gastrointestinal endoscopy showed a large, pedunculated polyp (3.5 cm in length), the base of which was located in the anterior wall of the duodenal bulb. Extensive erosions were seen on the top of the polyp, and the pedicle of the polyp had become twisted on its axis (Fig. 1A). Oozing hemorrhage from the area of the top in contact with the surrounding structures was found (Fig. 1B). The polyp except in the areas of erosion was covered with normal mucosa, and was considered to be non-epithelial. Grossly, polyps related to Brunner’s glands, GIST, and inflammatory fibroid polyp were suspected.

The patient was fasted, and placed on PPI therapy. Warfarin and LDA were discontinued, and heparin was administered. After improvement of the anemia and normalization of the PT-INR, heparin was withheld temporarily, and endoscopic polypectomy was performed.

**Figure 2:**
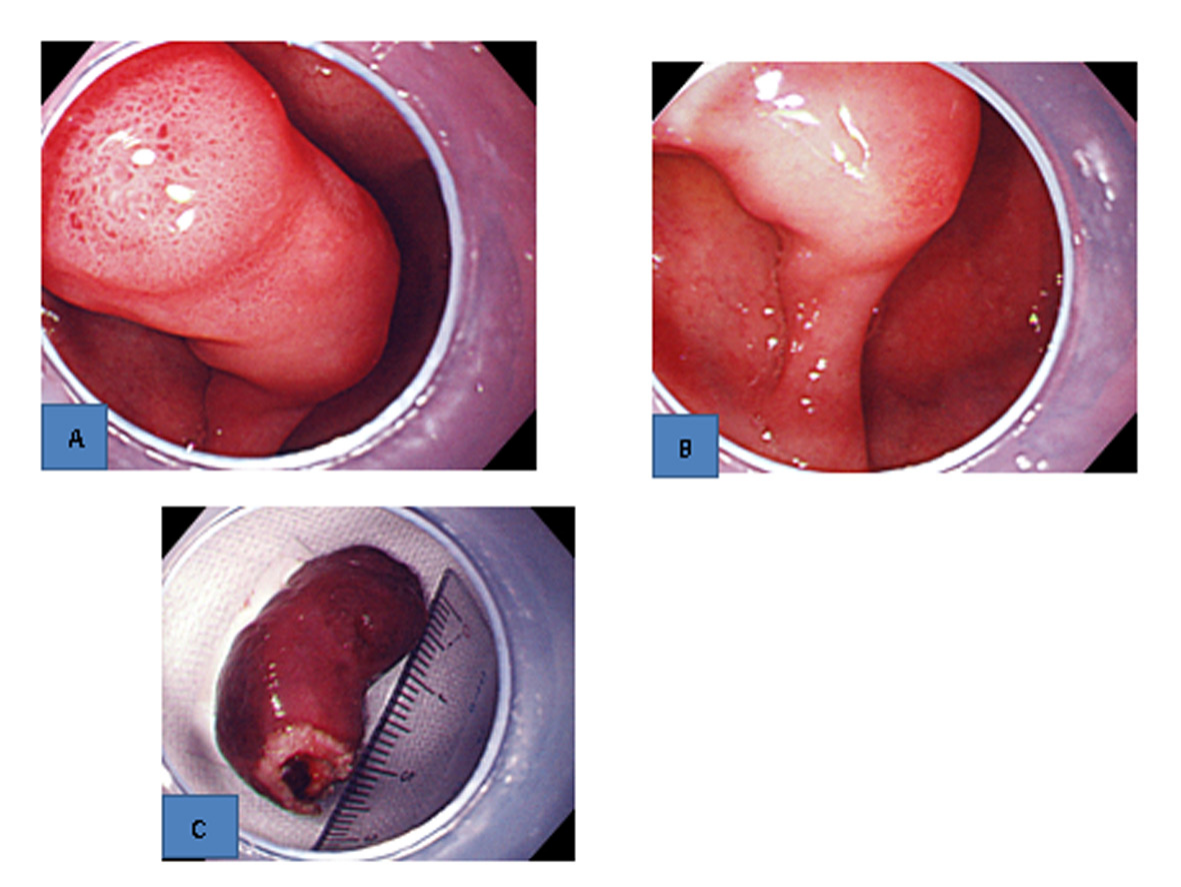
Upper gastrointestinal endoscopic findings. A). The bleeding from the erosions on the top stopped. B). The twisting of the pedicle was reduced. C). Resected specimen. A thick blood vessel was found in the pedicle.

Upper gastrointestinal endoscopic examination showed that the bleeding from the erosions on the top of the polyp had stopped (Fig. 2A), and the twisting of the polyp pedicle had been reduced (Fig. 2B). Since the pedicle was relatively thin, hemostatic clips were placed around its base, and snare polypectomy was performed.

**Figure 3:**
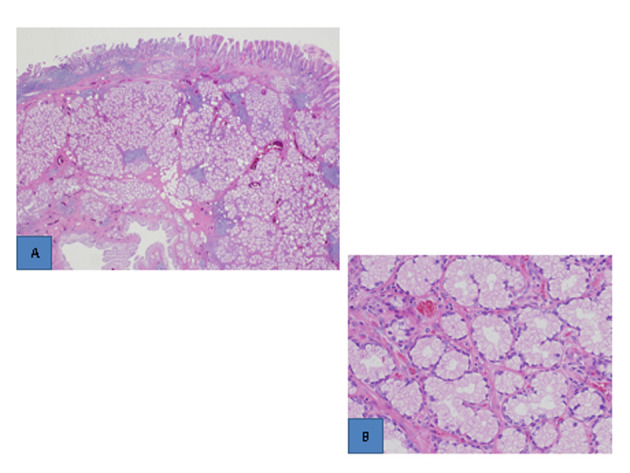
Histopathological findings (H.E. stain). A). The surface of the polyp was covered with an eroded epithelium, and hyperplastic Brunner’s glands were densely packed in the submucosal layer (low-power view). B). Atypia was not found (high-power view).

Although gross examination of the resected specimen revealed a thick blood vessel in the pedicle (Fig. 2C), no bleeding occurred during polypectomy. Histopathological examination showed that the surface of the polyp was covered with an eroded duodenal epithelium, and hyperplastic Brunner’s glands were densely packed mainly in the submucosal layer (Fig. 3A). Atypia was not found in the hyperplasia (Fig. 3B). These findings led to a diagnosis of Brunner’s gland hyperplasia.

No bleeding occurred after polypectomy, treatment with warfarin and LDA was resumed, and the patient was discharged.

## Discussion

Brunner’s glands exist in the duodenum, and secrete alkaline mucus. They are distributed densely in the duodenal bulb, but become progressively more thinly distributed toward the anal side. Lesions related to Brunner’s gland include hyperplasia, adenoma, and hamartoma. Since Brunner’s glands are located in the submucosa and deep layer of the lamina propria, these lesions present as submucosal tumors.

Brunner’s gland hyperplasia represents a nonneoplastic change, and occurs second-most commonly, after ectopic gastric mucosa among the elevated lesions of the duodenum. The proposed causes of Brunner’s gland hyperplasia include compensatory changes associated with pancreatic exocrine insufficiency [1] or with gastric hyperacidity [2], and an increase in pepsinogen II levels [3], but these have not been established. Most Brunner’s gland hyperplasia lesions are small and asymptomatic, and require no treatment, but, rarely, they enlarge and bleed. A search of Japan Medical Abstracts Society and MEDLINE for the 15-year period of 1996 - 2010 revealed 8 patients with bleeding Brunner’s gland hyperplasia in Japan [4, 5]. The clinicopathological features of hemorrhagic Brunner’s gland hyperplasia were analyzed in these 8 patients and our 1 patient (Table 1). They were mostly middle-aged or older men (over 40 years of age). All 9 patients had pedunculated polyps, 8 of which were large, with a diameter of 2 cm or more. Eight of them were located in the duodenal bulb, where Brunner’s glands are most heavily concentrated. Two patients took antithrombotic agents. Spontaneous and post-biopsy bleeding occurred in 7 and 2 patients, respectively, from erosions on the top of the polyp. In the present patient, the polyp was quite mobile, became twisted on its pedicle, was extensively eroded on the top, and bled from the area of the top in contact with the surrounding structures. Despite heparinization after hospital admission, bowel rest by fasting and PPI administration resulted in hemostasis. Thus, we speculate that the erosions were produced by the friction of the polyp against the surrounding structures, and that, although administration of antithrombotic agents may have contributed to bleeding, prolonged friction, congestion due to the twisting of the pedicle, and exposure to stomach acid, in combination, led to bleeding. Similarly, the other 8 patients had pedunculated polyps, which were mobile on their pedicles and susceptible to friction. Therefore, it was presumed that bleeding from Brunner’s gland hyperplasia occurred, not as a result of auto-destruction, but mainly due to mechanical injuries, such as scratches to the top of the pedunculated polyp. Most bleeding polyps became enlarged, and the polyp epithelium became stretched, weakened, and susceptible to injury, thereby presumably contributing to bleeding.

**Table 1:** Reported Cases of Bleeding Burnner’s Hyperplasia in Japan (1996 - 2010, Including Our Case)

Number of cases	2
Age: 40 y.o. ≥ / 40 y.o. <	7/2
Sex: male/female	7/2
Form: pedunculated polyp/others	9/0
Size: 2 cm ≥ / 2 cm <	8/1
Location: bulb/descending part	8/1
Antithrombotic drugs: (+)/(-)	2/7

On the other hand, when administering antithrombotic agents, attention should be paid to the presence, not only of ulcerative lesions, but also of bleeding-prone conditions such as in the present case.
